# A Critical Use of Foucault’s Art of Living

**DOI:** 10.1007/s10699-015-9441-z

**Published:** 2015-10-29

**Authors:** Marli Huijer

**Affiliations:** Faculty of Philosophy, Erasmus University Rotterdam, P.O.1738, 3000 DR Rotterdam, Netherlands

**Keywords:** Foucault, Ethical vocabulary, Problematization, Technology, Care of the self

## Abstract

Foucault’s vocabulary of arts of existence might be helpful to problematize the entwinement of humans and technology and to search for new types of hybrid selves. However, to be a serious new ethical vocabulary for technology, this art of existence should be supplemented with an ongoing critical discourse of technologies, including a critical analysis of the subjectivities imposed by technologies, and should be supplemented with new medical and philosophical regimens for an appropriate use of technologies.

In search for a new ethical vocabulary for technology, Steven Dorrestijn turns to the ancient Greek and Latin arts of living, as described by Michel Foucault. Rather than obeying moral laws or conforming to moral codes, Greek and Roman citizens shaped their morality in practices of the self: free male citizens set themselves rules of conduct, sought to transform themselves and to make their life a work of art. Morality was not pre-given, but came into being in self-practices. This interwovenness of self-practices and morality seems to fit well the current interwovenness of technology and morality. When we acknowledge that morality cannot be separated from technology, Dorrestijn is right to press us to reflect on how the hybrid morality/technology could be stylized in a manner that ‘carries certain aesthetic values and meets certain stylistic criteria’ (Foucault 1990a, 11).

But, what happens in this relocation of the ancient Greek and Greco-Roman arts of existence to current technological ways of living? Dorrestijn not only jumps through the millennia, he also relocates an ethics developed in the area of the desires (food, diet, sex) to the area of technology. Does the problematization of sexual activity, as expressed by philosophers and medical doctors in the Greek culture of the fourth century BCE and the Greco-Roman culture of the second century of our time, offer an adequate ethical vocabulary for technology? And does Dorrestijn take sufficiently serious the problematization, which the ancient Greeks and Romans took as starting point for the stylizing of the self?

In my response, I will focus on the Greek and Latin problematization, or moral concern, of the desires. Is this problematization suitable for our current dealings with technology? In the course of the centuries, knowledge and power systems have produced subjectivities unknown to Greek and Roman men. Resisting and refusing these subjectivities is now an important instrument in transcending oneself and shaping new subjectivities, as Foucault explained in interviews and articles. Shouldn’t we resist and sometimes refuse technologies to be able to aesthetically stylize our lives? And, is Foucault’s ethics as an art of existence not too individualistic to provide the ethical vocabulary that we need to critically reflect on or even refuse technologies?

## Problematization

In *The Use of Pleasure* and *Care of the Self*, volume 2 and 3 of *The History of Sexuality* (published in French in 1984), Foucault devoted himself to the manner in which ancient Greek and Latin culture problematized sexual activity. Questions guiding him were how, why and in what form sexuality was constituted as a moral domain and why this moral concern, this ‘problematization’, of sexuality was so persistent. What structured the moral experience of sexual pleasures? He aimed ‘to define the conditions in which human beings “problematize” what they are, what they do, and the world in which they live’ (Foucault 1990a, b, 10).

To explain how the Greeks and Romans constituted the *aphrodisia* (the acts, gestures and contacts that produce pleasure. In Latin: *venerea*) as a domain of moral concern, Foucault used four notions: (1) the ontology, (2) the deontology, (3) the ascetics and (4) the teleology of the moral experience of sexuality. In less philosophical terms: what is the force of sexual desires, and how does this force link together acts, pleasures and desires (ontology)? How to enjoy sexual pleasure ‘as one ought’ (deontology)? What attitude to oneself is required (ascetics)? What does the ethical subject, once fulfilled, look like (teleology)?

At the core of Greek and Greco-Roman thought, Foucault noticed fears and exigencies often seen as characteristic for the later Christian ethic: sexual activity was feared because of the potential exhaustion of the organism; mutual faithfulness among marriage partners was highly valued; love between men, though not condemned, was surrounded by many rules of conduct; and sexual abstention was seen as a form of mastery. Though sexual pleasure was in itself not an evil, sexual acts were regarded as dangerous, difficult to master, and costly (Foucault 1990b, 237). Hence the development of a fourfold thematic of sexual austerity around the body, marriage, relations between men and the existence of wisdom. All of these sexual regimens were aimed at stylizing a freedom, stylizing a subjectivity, in which sexual acts were integrated into the management of health and the life of the body (Foucault 1990a, 21, 97–98).

## From Antiquity to Today

‘Do you think that the Greeks offer an attractive and plausible alternative?’ Hubert Dreyfus and Paul Rabinow asked Foucault during a working session at Berkeley in April 1983. ‘No!’, Foucault replied, ‘I am not looking for an alternative; you can’t find the solution of a problem in the solution of another problem raised at another moment by other people’ (Foucault 1984, 343). Greek ethics was related to a virile society with slaves, and with women treated as underdogs. All ‘quite disgusting’, Foucault said. Yet, what he liked about the Greeks was their interest in problems or in dangers rather than in solutions: ‘I think that the ethico-political choice we have to make every day is to determine which is the main danger’ (Ibid., 343). The Greeks problematized desires, Kant problematized intentions, and in our society the main field of morality is our feelings, he suggested (Ibid., 352).

One of the few other texts in which Foucault connected the ancient Greek and Greco-Roman care of the self to modern ways of turning oneself into a subject, is ‘The Subject and Power’, his 1982 afterword to Dreyfus and Rabinow’s *Michel Foucault. Beyond Structuralism and Hermeneutics*. Interesting about the Greeks and Romans is that they could stylize themselves free from any social institutional system. That freedom is lost in the course of time. Nowadays human beings are permanently submitted to historically grown fields of knowledge and linked to systems of rules and constraints, which define, constitute and normalize the experience they have of themselves. In order to find new types of subjectivity, we therefore need to struggle against what Foucault called ‘the submission of subjectivity’. In his words:

Maybe the targets nowadays is not to discover what we are, but to refuse what we are. … We have to promote new forms of subjectivity through the refusal of this kind of individuality which has been imposed on us for several centuries (Foucault 1982, 216).

Creating one’s self as a work of art implies that one refuses given subjectivities, imposed by knowledge and power systems, and relates ‘the kind of relation one has to oneself to a creative activity’ (Foucault 1984, 351).

## Ethics in Times of Technical Mediation

What does Dorrestijn do when he relocates the ancient notion of problematization to our time?

To start, he defines ‘technological mediation and the hybridization of our existence’ as the material for problematization (ontology). His argument for this ‘ethico-political choice’ is that some philosophers fear that ethics will not survive the mediation approach. Does that mean that mediation and hybridization are the main danger, the main field of morality, of our society? That conclusion would be correct if Dorrestijn would not dismiss all talk of dangers. He distances himself from ‘second stage’ philosophers of technology like Heidegger, Ellul and others, who emphasized the dangers of technology. In contrast to them, Dorrestijn, as a third stage philosopher of technology, conceptualizes technology as something familiar, as part of our human existence. That leaves us in a difficult position. Why would we problematize technological mediation if it is not the main danger we experience today? Was Foucault right, and is the main field of morality in our society not technology, but feelings?

Second, Dorrestijn proposes a ‘critical ontology of our technically mediated existence’. This critical ontology is needed to find openings to possible transformations of our way of being. Although these phrases look like those of Foucault, Dorrestijn does not elucidate what this critical ontology consists of and how we can reach transformations of the self. Foucault’s analysis of Greek and Latin critical ontology of sexual desires is based on a large number of philosophical and medical texts that not only problematize the aphrodisia, but also suggest a whole regimen, or ‘dietetics’, which define the appropriate use of pleasure (that is, appropriate to the circumstances). These kind of philosophical and medical references are lacking in the description of Dorrestijn. If we take Foucault’s problematization seriously, we should scrutinize technology as meticulously as the Greeks and Romans scrutinized sexual acts. And like the Greek and Romans we would need medical and philosophical regimens, rules of conduct, which we can use to determine how we can enjoy technology ‘as we ought’.

Third, Dorrestijn takes Foucault’s art of living as an unproblematic good. Yet, Foucault’s presentation of Greco-ethics as an ethics of the pleasure one takes in oneself (Foucault 1990a, b, 66) is said to be inaccurate: the Stoics for example did not find joy in themselves but in going beyond the self and thinking and acting in harmony with universal reason (Hadot 1986, 207). Political philosophers, including Michael Walzer, Jürgen Habermas and Charles Taylor, have criticized Foucault for his anti-universalistic stance and his refusal to set up new rules or codes of conduct (Hoy 1986; Huijer 1999). The use of Foucault’s art of living seems to be limited to the individual who aims to make a work of art of his or her hybrid self and who is not interested in normative notions concerning social and environmental effects of technology.

In sum, Foucault’s vocabulary of arts of existence might be helpful to problematize the entwinement of humans and technology and to search for new types of hybrid selves. However, to be a serious new ethical vocabulary for technology, this art of existence should be supplemented with an ongoing critical discourse of technologies, including a critical analysis of the subjectivities imposed by technologies, and should be supplemented with new medical and philosophical regimens for an appropriate use of technologies. Critical reflection and new types of dietetics are an indispensable instrument, both at the individual and the social level, to reach human/non-human hybrids that meet certain aesthetic as well as social values. The option that individuals or society refuse certain technologies, such as nuclear technology, should be kept open.
